# A Miniaturized and Ultra-Low-Power Wireless Multi-Parameter Monitoring System with Self-Powered Ability for Aircraft Smart Skin

**DOI:** 10.3390/s24247993

**Published:** 2024-12-14

**Authors:** Chongqi Wang, Yu Wang, Wei Pu, Lei Qiu

**Affiliations:** Research Center of Structural Health Monitoring and Prognosis, State Key Laboratory of Mechanics and Control for Aerospace Structures, Nanjing University of Aeronautics and Astronautics, Nanjing 210016, China; wcqwcq@nuaa.edu.cn (C.W.); puwei2000@nuaa.edu.cn (W.P.)

**Keywords:** aircraft multi-parameter monitoring, energy self-supply, aircraft smart skin, miniaturized, structural health monitoring, ultra-low power consumption

## Abstract

The aircraft smart skin (ASS) with structural health monitoring capabilities is a promising technology. It enables the real-time acquisition of the aircraft’s structural health status and service environment, thereby improving the performance of the aircraft and ensuring the safety of its operation, which in turn reduces maintenance costs. In this paper, a miniaturized and ultra-low-power wireless multi-parameter monitoring system (WMPMS) for ASS is developed, which is capable of monitoring multiple parameters of an aircraft, including random impact events, vibration, temperature, humidity, and air pressure. The system adopts an all-digital monitoring method and a low-power operating mechanism, and it is integrated into a low-power hardware design. In addition, considering the airborne resources limitations, an energy self-supply module based on a thermoelectric generator (TEG) is developed to continuously power the system during flight. Based on the above design, the system has a size of only 45 mm × 50 mm × 30 mm and an average power consumption of just 7.59 mW. Through experimental validation, the system has excellent performance in multi-parameter monitoring and operating power consumption, and it can realize the self-supply of energy.

## 1. Introduction

The aircraft smart skin (ASS) is a revolutionary new technology that will change the future of aircraft design. It is a technology of embedding and integrating sensors, actuators, and microprocessors into aircraft skin structure to form an aircraft neural network during the design and manufacturing process so as to monitor, analyze, and process multiple physical parameters of the environment and the aircraft structures, in addition to adaptively controlling the corresponding performance of the aircraft [1,2,3,4].

The application of ASS is extremely important in cases such as structural shape optimization, aerodynamic load measurement, flight state prediction, and structural health monitoring. It can provide the health status of the aircraft structure itself and monitor the service environment. The key to the problem lies in the need to integrate various sensors with different functions into the aircraft skin structure and to develop a multi-parameter, multi-channel data acquisition and monitoring system.

Sensing ability is the foundation of ASS implementation. Flexible printed circuit (FPC) technology as a fabrication process with long-term development, has been applied by many scholars to develop multi-parameter sensors for ASS. Yang et al. [5] prepared copper-based capacitive sensors using a polyimide (PI) substrate, developing a lightweight, multi-parameter ASS for aircraft composite materials. This smart skin can monitor the temperature distribution of composite material structures in real-time during the manufacturing process, as well as the structural health status during usage. Suzuki et al. [6] proposed a large ASS, with the basic unit being a lightning protection shield sensor based on a flexible resistive sensitive layer. When the structure is damaged by impact, its potential changes, which allows for the detection of damage and is used for the lightning protection and damage localization of the skin structure. Dudley et al. [7] developed a structural health monitoring (SHM) ASS based on a flexible, lightweight, and large-scale SansEC sensors array, designed for in situ damage detection and diagnostics of aerospace composite materials, effectively identifying internal damage within composite structures.

Many scholars have also carried out the development of an ASS multi-parameter monitoring sensor network. Chang and Qiu et al. [8,9], respectively, developed a kind of flexible smart piezoelectric layer based on a large area flexible printed circuit fabrication process and the piezoelectric sensor (PZT) encapsulation process, which can realize the damage and impact monitoring of the aircraft skin structure. Xiong et al. [10] proposed an intelligent flexible sensing skin that consists of capacitive sensors, piezoelectric sensors, thermal film sensors, temperature sensors, and strain sensors integrated into the aircraft skin structure, and it is capable of monitoring of a wide range of aircraft structural multi-parameters such as pressure, temperature, wall shear stress, the flutter vibration of the surface of the wing skin, and the position of sudden impacts. Wang et al. [11] proposed a lightweight stretchable flexible piezoelectric sensing network capable of scaling up to 2500% of the original area to enable the monitoring of damage and impact of large-area aircraft skin structures. In addition, Kopsaftopoulos et al. [12] proposed a stretchable sensor network consisting of a variety of micro-sensors, including piezoelectric, strain, temperature, and pressure sensors. These sensor networks were integrated into the wing composite layer, giving the aircraft the ability to sense the multi-parameter health state of the structure.

In addition, processes such as inkjet printing and screen printing are also important ways for achieving ASS. Mascareñas et al. [13] sprayed graphene oxide (GO) on cellulose paper and embed it in composite structures to develop a bio-inspired smart skin based on a flexible capacitive sensor array, which can be used for the crack monitoring of skin structures. Zhao et al. [14] used aerosol jet printing technology to print nano-silver particle ink directly onto carbon fiber prepreg and coat it with insulating epoxy resin film to form a strain sensor pattern, realizing an aircraft composite material smart skin with an intrinsic strain sensing capability. Blumenthal et al. [15] researched the fabrication process of a direct print circuit system using aerosol jet printing on a complex shape surface for the stress monitoring of cut sections. This work is of great significance for the realization of lightweight aircraft smart skin based on structure-sensing integration.

However, the sensors and sensor networks reported above still need to be connected to external multi-parameter monitoring systems for sensor signal acquisition, transmission, and processing to achieve multi-parameter sensing. For airborne applications of ASS monitoring systems, many scholars have carried out relevant research. Qiu et al. [16] developed a composite structure impact monitoring system suitable for large-area piezoelectric sensor networks, and they reduced the volume and power consumption of the system through hardware design and an area-only localization monitoring method. Krichen et al. [17] proposed a wireless sensing network architecture for aircraft wing vibration monitoring, which consists of a wireless sensing network deployed on a structure such as the wing and multiple node systems for structural state sensing, data relay, and data analysis. Kim et al. [18] developed an aircraft health and usage monitoring system that is capable of monitoring strain data during flight using fiber optic sensors embedded in the main wing structure. Kwon et al. [19] developed an aircraft wing load monitoring system, which could acquire the strain distribution and other flight parameters through the integrated fiber Bragg grating sensors in the aircraft’s composite wing structure, realizing the monitoring of the loads on the aircraft wing structure at all flight stages. Due to the limitations of airborne resources, the ASS monitoring system must follow strict development requirements in terms of volume, weight, and power consumption.

However, the above monitoring systems have a single function, so they need several monitoring systems to monitor the multiple parameters of an aircraft. In addition, they are mostly powered by power lines or batteries, which to a certain extent limits the development of lightweight and low-power consumption airborne monitoring systems, rendering them not conducive to the realization of ASS.

In recent years, energy harvesting technologies have been extensively studied, mainly with regard to kinetic energy harvest, solar energy harvest, and thermal energy harvest [20,21,22,23]. Among them, thermal energy harvest technology has a great advantage in the application of ASS, which can use the temperature difference in the aircraft skin structure during different flight stages for energy harvesting [22,23]. Elefsiniotis et al. [24] fixed a thermoelectric generator (TEG) on the fairing of an aircraft engine installation tower, and they realized thermal energy harvest by using the temperature difference between the inside and outside of the fairing. Samson et al. [25] combined TEG with an energy storage unit to power wireless sensor nodes under the specific external temperature change in the aircraft. Featherston et al. [26] used the temperature difference between the fuel tank of the aircraft wing and the outside atmosphere to generate electricity to power the developed wireless sensor network. This provides solution ideas for the energy supply of multi-parameter monitoring systems.

Considering the need for multi-parameter monitoring in the realization of ASS, this paper presents the development and evaluation of a miniaturized and ultra-low-power wireless multi-parameter monitoring system (WMPMS) with self-powered ability. The highlights of the system are as follows: (1) By adopting an all-digital monitoring method, the hardware of the system is greatly simplified. With an overall dimension of 45 mm × 50 mm × 30 mm, the system is capable of monitoring impact events, vibration, temperature, humidity, and air pressure online. (2) By adopting low-power hardware design and the corresponding low-power working mechanisms like sleep–wake-up, the average power consumption of the device is only 7.59 mW. (3) A TEG-based energy self-supply module is also developed, which enables the WMPMS to continuously work onboard for 11 h without an external energy supply. (4) In addition, due to the integrated wireless communication module, the system has the ability to cooperate with other systems to achieve the large-scale, multi-part monitoring of the aircraft.

The rest of this paper is organized as follows: In Section 2 the basic principles of the IoT-based aircraft multi-parameter monitoring method is proposed. Section 3 describes the design of the multi-parameter monitoring system, followed by the development of the energy self-supply module in Section 4. Section 5 verifies the multi-parameter monitoring function of the system, as well as its power consumption and self-power supply ability. Finally, Section 6 presents some conclusions.

## 2. Basic Principles of ASS-Based Aircraft Multi-Parameter Monitoring

### 2.1. ASS Multi-Parameter Monitoring Architecture

**Figure 1:**
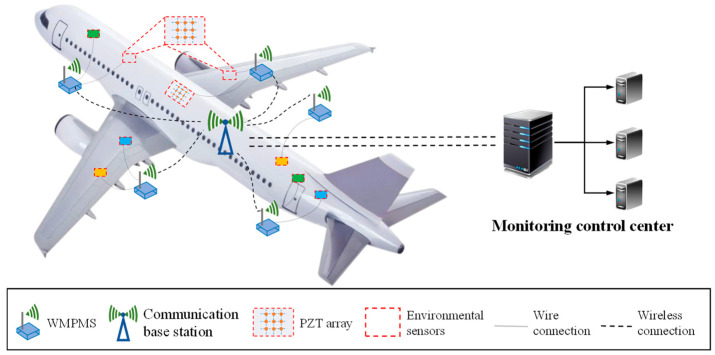
Architecture of ASS-based multi-parameter monitoring of an aircraft.

The overall architecture of the multi-parameter monitoring of ASS is shown in Figure 1. This architecture mainly contains sensor networks, WMPMS, a communication base station, and a monitoring control center [27]. The proposed architecture can be used to collect essential information about the structural health status and service environment, and it is thus capable of guiding maintenance and ensuring flight safety.

By connecting various sensors placed on the aircraft structure, the WMPMS is able to monitor impact events, vibration, temperature, humidity, and air pressure online. Since the large-scale monitoring of multiple parts of the aircraft structure is usually required, several monitoring systems distributed at different locations are necessary. After obtaining the multi-parameter monitoring results, each system transmits its respective monitoring parameter results to a communication base station and forwards them to a monitoring control center which is responsible for storing, processing, analyzing, and making decisions based on the information.

### 2.2. All-Digital Multi-Parameter Monitoring

In order to realize the miniaturization and low-power consumption of the proposed system, an all-digital multi-parameter monitoring method is presented in this paper to simplify the hardware system and enable the monitoring of impact events and the aircraft service environment. The all-digital multi-parameter monitoring method mainly consists of two parts, namely the digital sequence-based impact region localization method and the digital sensors-based service environment monitoring method.

The impact monitoring of aircraft structures is a typical passive structure health monitoring method. A monitoring method based on piezoelectric-guided waves is widely used because of its high sensitivity and regional monitoring. When an external impact occurs on the surface of the structure, a stress wave (defined as impact signal) is generated and propagated from the impact location to the perimeter of the structure. An impact signal is a kind of guided wave signal, and its energy decreases as the propagation distance increases. So, the closer the PZT sensor feels its position is in relation to the impact, the stronger the guided wave energy, and the greater its response will be. And in general, the closer sensors receive the impact signal earlier. By analyzing the arrival sequence and energy size of the impact signals obtained by PZT sensors at different positions, the impact monitoring results are obtained by combining a specific algorithm [28,29].

**Figure 2:**
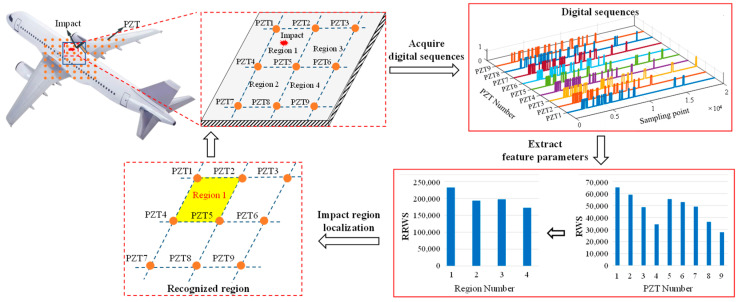
Basic principle of the digital sequence-based impact region localization method.

On this basis, this paper proposes an impact region location method based on digital sequence, the basic principle of which is shown in Figure 2. Different from the conventional guided wave-based impact monitoring methods [30], this method converts the analog impact response signals of PZTs directly into digital sequences, which only consists of high level ‘1’s and low level ‘0’s. By extracting digital characteristics from the sequences, the impact region, which is normally surrounded by four PZTs, can be recognized with a simple localization algorithm. Since the analog impact response signals are converted into digital sequences in the front end, all the corresponding complex analog circuits like data acquisition and signal conditioning can be removed. Therefore, the hardware system can be greatly simplified, making it possible to achieve a small size and low power consumption.

To locate the impact region, the above-mentioned digital localization algorithm first extracts a digital signal characteristic named reverse weighted sum (*RWS*) from the digital sequence of every PZT. The definition of *RWS* is given by Equation (1), which is used to fuse the sampling time and digital level of the sequence, so that the impact-induced influence on PZTs can be reliably described. Since the PZT nearest to the impact position is the first sensor that receives the impact caused by the guide waves and outputs the impact response signal, it should have the largest *RWS* value. Then, the impact-induced influence on the region can be calculated by summing up the *RWS* values of its four PZTs, as shown by Equation (2). After calculating the *RWS* values of every region, the region with the largest value can be recognized as the impact region.

(1) $$RWS\left(\mathrm{PZT},r\right)=\sum_{j=1}^{s}\left[(s+1-j)\times W_{j}\right]$$

(2) $$RWS\left(\mathrm{Region},p\right)=\sum RWS\left(\mathrm{PZT},r\right)$$

where *r* is the PZT number, *s* is the length of the digital sequence, *j* is the serial number of the sampling point in the digital sequence, *W_j_* is the digital value corresponding to the *j*-th sampling point, and *p* is the region number.

The basic principle of the digital sensor-based service environment monitoring method is proposed, and it can realize the monitoring of different environmental parameters by placing sensors at key parts of aircraft structures. Compared with conventional analog sensors, the size and power consumption of digital sensors can be greatly reduced by adopting micro electromechanical systems (MEMS)-based manufacturing technology. Therefore, this paper adopts digital sensors to monitor multiple important environmental parameters of aircraft, including vibration, temperature, humidity and air pressure. The adopted digital sensors can realize the sensing, processing, storage, and digital outputting of the environmental parameters [31,32]. In addition, due to the advantages of high expansibility and low power consumption, the widely used inter-integrated circuit (I2C) bus is adopted to enable signal transmission between digital sensors and the developed multi-parameter monitoring system [33].

## 3. Design of the WMPMS

In this section, the design and implementation of the proposed WMPMS are described in detail in two aspects, hardware and software.

### 3.1. Hardware Design

**Figure 3:**
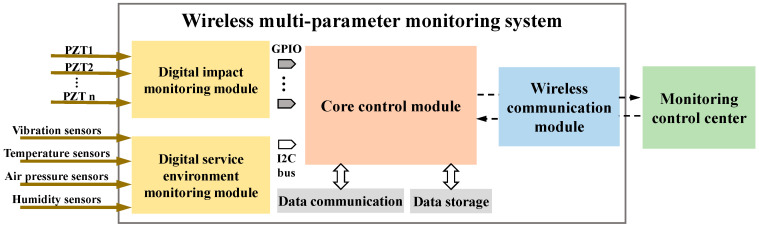
Overall hardware architecture of the WMPMS.

The hardware architecture of the WMPMS is shown in Figure 3, which contains four modules, including a digital impact monitoring module, a digital service environment monitoring module, a core control module, and a wireless communication module. The digital impact monitoring module is connected to the PZT array, converts the impact response signals into digital sequences, and uploads them to the core control module. The digital service environment monitoring module is connected to digital sensors and uploads the obtained environmental signals to the core control module. The core control module uses the Apollo 2 microcontroller with ARM cortex M4 core, which has high integration and ultra-low power consumption compared to FPGA and DSP. It is responsible for collecting multi-parameter monitoring signals, calculating monitoring results, and storing and transmitting them to the monitoring control center through the wireless communication module.

Also, the digital impact monitoring module digitizes the impact response analog signal through the transistortransistor logic (TTL) pin logic level standard of the core processing chip. In the WMPMS, the impact response signal is converted to a high level ‘1’ when it is greater than 1.4 V and to a low level ‘0’ when it is less than 1.4 V. Prior to this, the impact response analog signal from the PZTs is limited by the Zener diode to less than 3.3 V. Compared with the conventional A/D conversion and comparator digitization conversion methods [34], the digital method based on TTL can effectively reduce the complexity of the hardware circuit and reduce the power consumption of the system.

As for the service environment monitoring, three different kinds of MEMS sensors are adopted, including the BMA250 sensor for vibration monitoring, the BMP280 sensor for air pressure monitoring, and the SHT30 sensor for temperature and humidity monitoring. MEMS technology can integrate microstructures, various sensitive micro-components, signal processing, and control circuits and communication interfaces on a chip, which has the advantages of miniaturization and low power consumption. The digital service environment-monitoring module communicates with these sensors through the I2C bus, including setting monitoring parameters and the reception of data, etc. The communication mode of the I2C bus and the diversity of MEMS sensors for environmental monitoring make the digital service environment monitoring module highly scalable. According to different parameter monitoring ranges and parameter types, the multi-parameter monitoring system can flexibly adapt to different monitoring scenarios.

The wireless communication module is also an important part of the WMPMS, which receives commands and transmits monitoring results to the monitoring control center. In order to realize low power consumption and the networking of multiple systems, the ZigBee technology was adopted to perform wireless communication in this paper. Compared with the Wi-Fi and Bluetooth communication protocols, ZigBee has the advantages of a longer communication distance and higher reliability [35,36].

**Figure 4:**
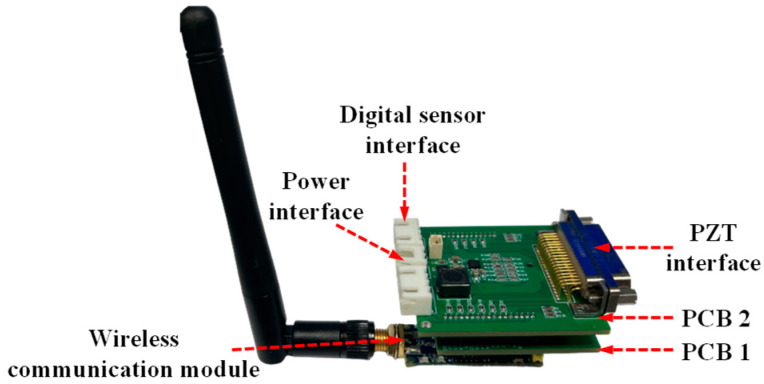
The developed WMPMS.

The developed WMPMS is shown in Figure 4, and it consists of two layers of PCBs and wireless communication modules. PCB 1 mainly integrates the core control module, and PCB 2 integrates the digital impact monitoring module, digital business environment monitoring module, PZT interface, digital sensor interface, and power supply interface. The two PCBs and the wireless communication module are connected to each other by row pins. Without the wireless antenna, the overall size is 45 mm × 50 mm × 30 mm.

### 3.2. Software Design

**Figure 5:**
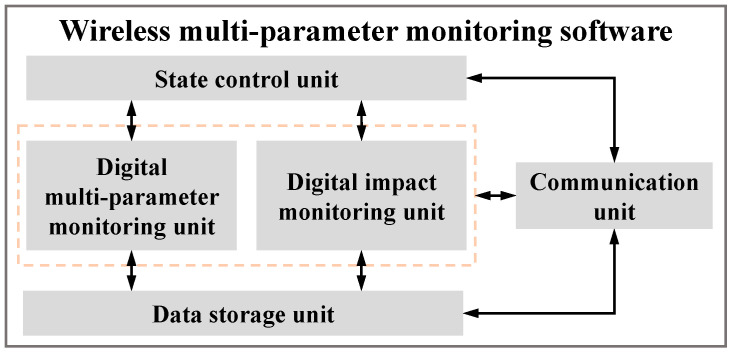
Software architecture of the system.

Based on the hardware design of the WMPMS, the corresponding embedded software is also developed to control the system, including switch of working state, signal acquisition, processing, storage, communication, etc. Among the multiple parameters that the system monitors, impact is a random event whose monitoring uses a triggering mechanism, and when an impact event occurs, the system is triggered to respond. As for environment monitoring, apart from vibration that should be continuously monitored, temperature, humidity, and air pressure can be monitored in fixed time intervals. According to the function requirements mentioned above, the software of the multi-parameter monitoring system contains five components, including a state control unit, a digital impact monitoring unit, a digital service environment monitoring unit, a data storage unit, and a and communication unit, as shown in Figure 5.

The state control unit relies on an interrupt setting to switch the working state of the WMPMS. After power on, the system enters sleep state to reduce power consumption. When an impact event occurs on the structure, the GPIO interface of the MCU is triggered by the impact response signals of the PZTs. Then, the state control unit switches the system on so as to wake up from the sleep state and starts to execute the interrupt service program for performing digital impact monitoring. Similarly, once the unit receives the specific timer interrupt, it drives the system to wake up and carry out the environment monitoring of digital sensors.

The digital impact monitoring unit is used for the PZTs. When triggered by an impact event, the unit digitizes the PZT analog impact response signal and calculates the *RWS* values for all PZTs. The multi-parameter monitoring unit is used for other digital sensors and is responsible for data acquisition, computation, and transmission from these digital sensors. All monitoring data are transmitted to a data storage unit.

The communication unit is responsible for the command and data transmission between the WMPMS and the monitoring control center, and it adopts the universal asynchronous receiver/transmitter (UART) method.

## 4. Development of the Energy Self-Supply Module

Due to the limitations of the airborne resources, in addition to the need to optimize the operating power consumption design of the monitoring system, the potential energy in the environment while the aircraft is in flight can also be fully utilized. In this paper, a TEG-based energy self-supply module is developed to power the WMPMS, so that the system is capable of continuously working onboard without an external energy supply. The basic principle is to use a semiconductor TEG to convert the significant temperature variation during different flight stages of an aircraft in flight into electric energy and output that to the system in a stable way. The overall architecture of the energy self-supply module is proposed, which mainly contains a thermoelectric conversion unit and a power management unit.

**Figure 6:**
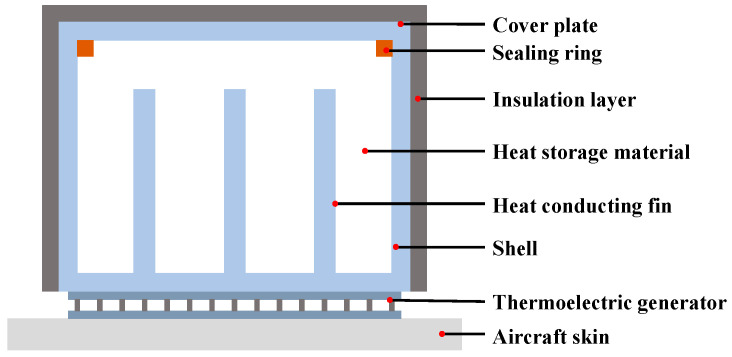
The thermoelectric conversion unit.

The architecture of the thermoelectric conversion unit is presented in Figure 6, which can be divided into a TEG and a heat storage structure. The upper end and lower end of the TEG are connected to the heat storage structure and the aircraft skin, respectively. When there exists temperature difference between the two ends of the TEG, it can generate electric power. Therefore, the key to thermoelectric conversion is ensuring a temperature difference between the heat storage structure and the aircraft skin exists.

When an aircraft is flying at cruise altitude, the skin temperature of the aircraft can be calculated using Equation (3).

(3) $$T_{R}=T_{S}\left(1+\frac{k-1}{2}M^{2}\times P_{r}^{\frac{1}{3}}\right)$$

where *T_S_* is the static atmosphere temperature at the flight altitude. Moreover, *k* is the specific heat ratio of air, the value 1.4. *P_r_* is the Prandtl number, and the value 0.7. *M* is the flight Mach number of the aircraft.

Assuming that the static atmosphere temperature of a civil aircraft at a normal cruise altitude is −40 °C and flight speed is Mach 0.8, then, the actual skin temperature is calculated to be −4.5 °C based on Equation (3). Similarly, the skin temperature is about 31.5 °C when the aircraft is at the take-off or landing stage. The temperature variation in aircraft skin temperature can reach 36 C. By selecting material with a large specific heat capacity and filling the heat storage structure, the above-mentioned temperature variation can be converted into the temperature difference between the heat storage structure and the aircraft skin.

In this paper, water is preliminarily adopted as the heat storage material since it has a large specific heat capacity and is easy to deploy. The thermoelectric conversion unit is shown in Figure 6. In this unit, a sealing ring is designed to prevent liquid leakage from the heat storage structure. In addition, several heat conduction fins are designed inside the shell to effectively increase the contact area between the heat storage material and the shell so that heat harvest efficiency can also be improved.

Based on the electric energy that the thermoelectric conversion unit generates, the power management unit is further designed to realize a stable and applicable voltage output for powering the multi-parameter monitoring system. Two main issues concerning the voltage rectifier and voltage management are solved by using this unit.

During the take-off stage, the temperature of the heat storage structure is higher than that of the aircraft skin, while the opposite occurs during the landing stage. Under these circumstances, the direction of the voltage that the TEG generates will also be opposite at the take-off and landing stages. Aiming to resolve this, a rectifier circuit based on a metal-oxide-semiconductor field-effect transistor (MOS) is incorporated into the design of the power management unit to maintain the direction of the voltage. Through performance tests, the voltage attenuation of the designed rectifier circuit is found to be only 5% and the power loss is only 9.8%, which are greatly reduced compared with the conventional diode rectifier bridge circuit.

As mentioned above, the output voltage of the thermoelectric conversion unit depends on the temperature difference in its two ends. Due to the instability of the temperature variation of the aircraft, the voltage amplitude is also unstable and cannot be directly used. Aiming to resolve the above issues, the power management unit is designed and integrated into the WMPMS. In this unit, a rechargeable battery is connected to regulate and harvest electrical energy. When the energy harvesting power of the thermoelectric conversion unit is greater than the power consumption of the system, the circuit controls the storage of excess power to the rechargeable battery. Otherwise, the battery switches to a discharged state and supplies power to the system through the hot spot conversion unit. In this way, stable and continuous power supply can be achieved.

## 5. System Verification

In this section, experiments are carried out to fully verify the developed WMPMS, including impact and environmental monitoring verification, system power consumption evaluation, and energy self-supply performance evaluation.

### 5.1. Multi-Parameter Monitoring Function Verification

#### 5.1.1. Impact Monitoring Verification

**Figure 7:**
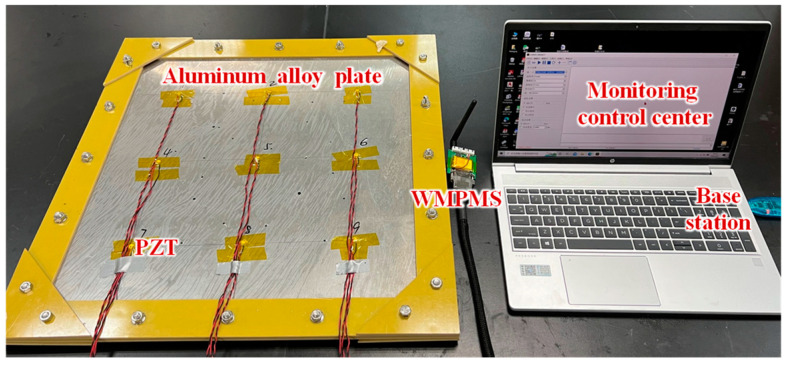
Experimental setup of impact monitoring.

The impact monitoring verification is performed on an aluminum alloy plate with dimensions of 600 mm × 600 mm × 3 mm (Length × Width × Thickness), as shown in Figure 7. A total of nine PZTs numbered from 1 to 9 are arranged on the structure and connected to the WMPMS. The four impact monitoring regions are formed, and the size of each region is 200 mm × 200 mm. During the verification process, 50 impact events are equally applied across the four regions by an impact hammer. Once an impact occurs, the system is awakened, converting the impact response signals of the nine PZTs into digital sequences, locating the impact region, and uploading the localization results to the monitoring control center through wireless communication, which is simulated by a laptop with a base station.

**Figure 8:**
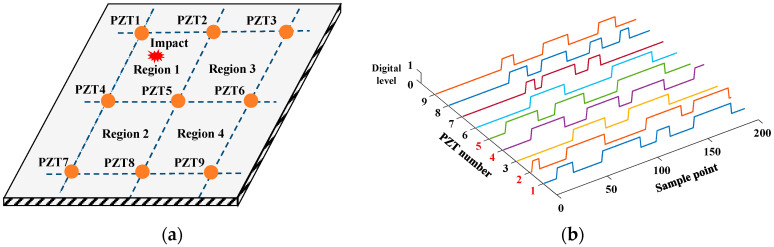
Example of impact monitoring: (**a**) impact occurring in region 1; (**b**) digital sequences.

Taking region 1 as an example, this region is surrounded by PZT1, PZT2, PZT4, and PZT5, as shown in Figure 8a. When impact occurs in this region, the digital sequences of all nine PZTs can be obtained by the system, as shown in Figure 8b. The PZTs’ *RWS* are calculated by taking the first 100 sampling points in the digital sequence, and according to Equation (1), shown in Section 2, the *RWS* values of PZT1, PZT2, PZT4, and PZT5 are much larger than those of the other PZTs, as shown in Table 1. Therefore, region 1 has the maximal *RWS* value among the four regions, as shown in Table 2. Then, region 1 is considered the impact region by the system, which is consistent with the actual situation. Among the applied 50 impact events, a total of 47 impact events are correctly located, and the accuracy rate of impact region localization is 94%. Therefore, the impact monitoring function of the developed WMPMS is verified successfully.

#### 5.1.2. Environmental Monitoring Verification

**Figure 9:**
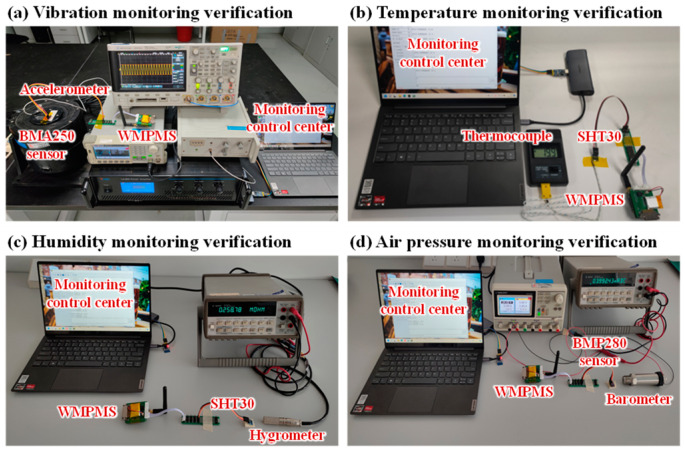
Experimental setup of environmental monitoring verification.

The experimental setup of the environmental monitoring verification of the developed WMPMS is shown in Figure 9. The verified parameters include vibration, temperature, humidity, and air pressure. Each parameter is monitored separately, and the monitoring results are obtained by the WMPMS and transmitted to the control center wirelessly.

**Figure 10:**
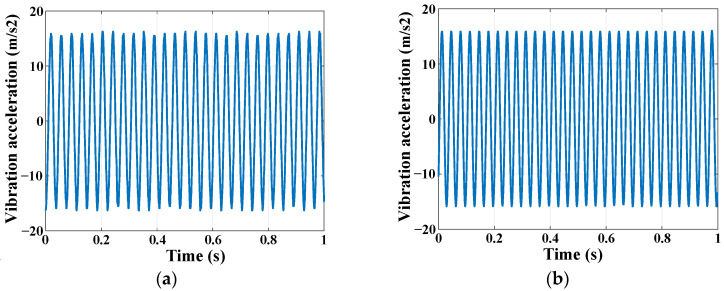
Vibration monitoring results: (**a**) WMPMS; (**b**) accelerometer.

As shown in Figure 9a, an arbitrary waveform generator is adopted to generate a sinusoidal signal with a frequency of 30 Hz, which is further amplified by a power amplifier and then causes a vibrostand to generate vibration. The digital vibration sensor BMP250 is placed on the vibrostand and connected to the WMPMS through the I2C bus to acquire the vibration signal. As a contrast, an accelerometer is also placed on the vibrostand and connected to an oscilloscope. Figure 10 shows the vibration monitoring results of the multi-parameter monitoring system and the accelerometer. Frequency spectrum analysis shows that the central frequency of the vibration monitoring result is about 30 Hz. And the peak-to-peak value of the acceleration amplitude is 15 m/s^2^, which is basically consistent with the test results of the accelerometer. However, by comparing Figure 10a,b, it can be seen that the monitoring results of the system cannot completely reproduce the sinusoidal vibration waveform due to the limited sampling rate of BMP250. However, the aim of aircraft smart skin vibration monitoring is to obtain the frequency and amplitude of the vibration, and the developed WMPMS can effectively obtain these two key parameters. For some of the more complex vibration monitoring scenarios, such as high sampling rate and multi-point monitoring, WMPMS can also achieve hardware expansion—expansion of sensors and sensing channels—and it can develop software to meet the monitoring requirements. In summary, the capability of the vibration monitoring of the developed WMPMS is verified.

**Figure 11:**
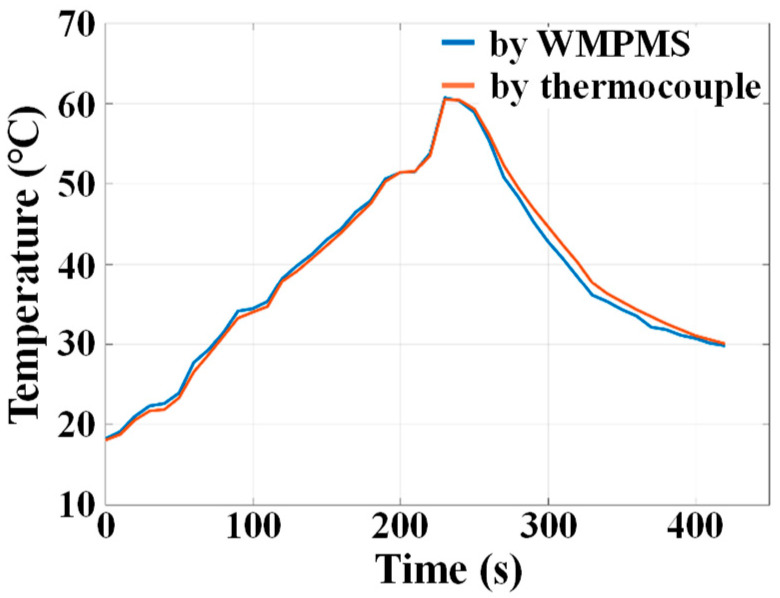
Temperature monitoring results.

The experimental setup of the temperature monitoring verification is shown in Figure 9b, in which the WMPMS connects a digital temperature/humidity sensor SHT30 to monitor the environmental temperature, and a thermocouple temperature sensor is adopted as a contrast. During the experiment, the SHT30 sensor and the thermocouple are first heated slowly from room temperature to about 60 °C using a heat gun, then, they are cooled back to room temperature. Figure 11 shows the temperature variation curves of the system and the thermocouple, which basically overlap with each other and represent the accuracy of the temperature monitoring of the WMPMS.

**Figure 12:**
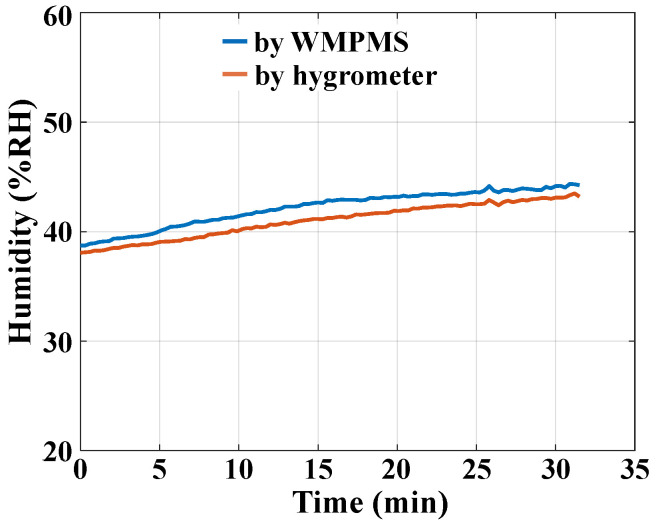
Humidity monitoring results.

As for the humidity monitoring verification, a hygrometer is placed together with the above-mentioned SHT30 sensor, and it is used as a reference, as shown in Figure 9c. During the experiment, the environmental humidity is controlled to rise slowly by using an air conditioner. Along with the humidity rise, the monitoring results of the system and the hygrometer are both recorded every 15 s, as shown in Figure 12. The monitoring deviation between the system and the hygrometer is about 1%RH, and the maximal relative error is only 2.4%, which is normally acceptable when performing humidity measurements with different sensors. Therefore, the developed WMPMS is capable of reliably monitoring environmental humidity.

**Figure 13:**
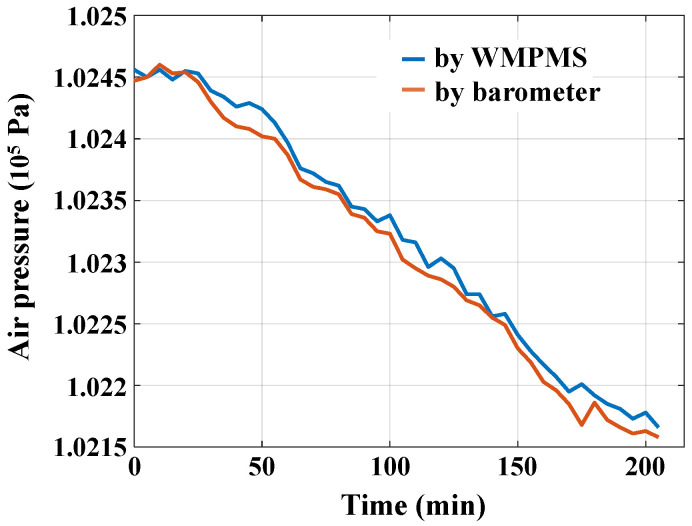
Air pressure monitoring results.

Finally, the air pressure monitoring verification is performed. As shown in Figure 9d, the WMPMS is connected to a digital air pressure sensor BMP280 to monitor pressure variation. Similarly, a standard barometer is adopted as a reference. During the experiment, both the multi-parameter monitoring system and the barometer record the current air pressure every 5 min. The whole monitoring process lasts for about 4 h, and the monitoring results are given in Figure 13. The experimental results show that the maximum deviation of air pressure values monitored by the system and the barometer does not exceed 22 Pa, which verifies the ability of the developed multi-parameter monitoring system to accurately monitor air pressure.

### 5.2. System Power Consumption Evaluation

In order to evaluate the power consumption of the WMPMS, a lithium battery is used to power the system, the service voltage of which is 3.3 V. By measuring the current of the system at different working states, the corresponding power consumption can be calculated, as shown in Table 3.

When the system is in sleep mode, the current is only 0.86 mA; therefore, the static power consumption is only 2.84 mW. Table 3 also gives the current values of different working modes, including impact monitoring, vibration monitoring, temperature and humidity monitoring, air pressure monitoring, and wireless communication. It can be seen that the system works with low power consumption when performing multi-parameter monitoring.

As for the power consumption assessment of wireless communication, the wireless communication module is only activated to transmit data in two cases. The first case is for the transmission of impact monitoring data when the structure is subjected to external impact, the system begins to collect and process the impact response digital signal, and the monitoring results are transmitted to the upper computer through the wireless communication module. The other case is for the service environment monitoring data transmission. In the embedded program of the system, the communication time interval is set, and when the set time interval is reached, the system uploads the environmental monitoring parameters to the upper computer. When no impact occurs and the set environmental monitoring data upload time is not reached, the system goes into sleep mode and continuously monitors the vibration state of the structure but does not transmit data. In addition, the amount of data transmitted by wireless communication in this paper is limited; the transmission speed can reach 250 kb/s, and each transmission process lasts only a few milliseconds. Therefore, although the working current of wireless communication is relatively high and reaches 28.06 mA, its duration is short when wireless communication is carried out, and the influence of the power consumption of wireless communication on the overall power consumption of the system is quite small.

In this paper, the time interval of service environment monitoring data communication is set to 10 s, and the impact is assumed to occur every 10 s, which is quite frequent; the duration time of every working mode is estimated and given in Table 3. According to the actual test results, the duration of impact monitoring and every kind of environmental parameter lasts less than 0.1 s. In addition, considering the working mode conversion of the wireless communication module, the whole communication process lasts less than 0.1 s. Therefore, the average power consumption of the system within a time period of 10 s can be calculated, and it is about 7.59 mW.

### 5.3. Energy Self-Supply Verification

**Figure 14:**
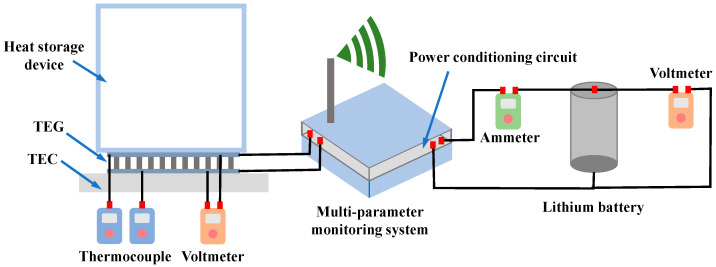
Schematic diagram of the energy self-supply verification setup.

**Figure 15:**
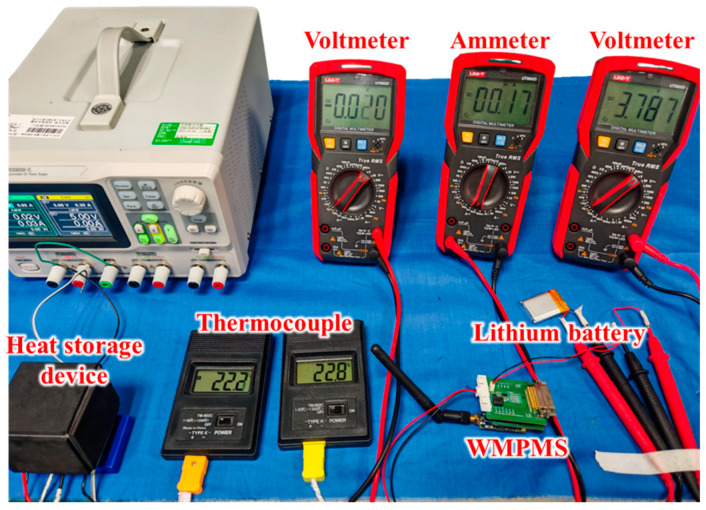
Physical diagram of the energy self-supply verification setup.

In this section, the feasibility of the energy self-supply module is verified, and the output power is analyzed and calculated. To simulate the temperature variation process of the aircraft wing during flight, including take-off, flight, and landing, a thermoelectric cooler (TEC) is adopted to control and change the temperature of the thermoelectric generator, by which the output voltage can be generated. The schematic and physical diagram of the experimental setup are shown in Figure 14 and Figure 15.

In Figure 14, the thermoelectric generator is placed on top of the TEC so that the lower surface of the thermoelectric generator can be heated or cooled by powering the TEC and changing the voltage direction. Two thermocouples are used to measure the temperatures of the lower surface and the upper surface of the generator, respectively. The output voltage of the thermoelectric generator is measured by a multimeter.

**Figure 16:**
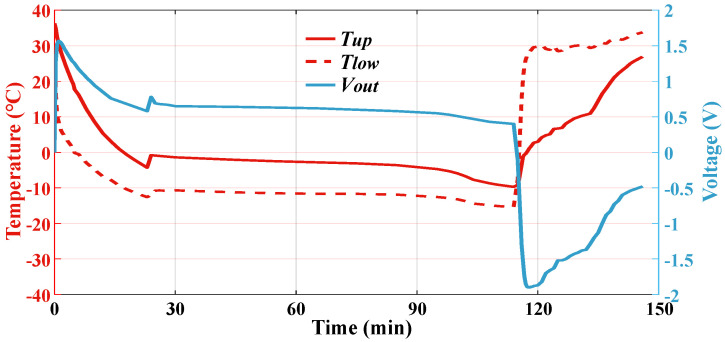
Verification results of the thermoelectric generator.

During the verification, the temperature of the thermoelectric generator’s lower surface, which is labeled as *T_low_*, is controlled to gradually decrease from room temperature to about −10 °C and then increase to about 30 °C. Figure 16 shows the changing process of *T_low_*, the temperature of the upper surface *T_up_* of the thermoelectric generator, and its output voltage *V_out_*. It can be seen that when the TEC starts to cool, *T_low_* decreases rapidly, while *T_up_* changes much slower due to the heat storage material. Therefore, *T_up_* is higher than *T_low_*. At the same time, *V_out_* starts to grow from zero, since the value of *V_out_* depends on the temperature difference between *T_up_* and *T_low_*. After about 110 min of cooling, the TEC switches to heating mode and *T_low_* increases rapidly, making the difference between *T_up_* and *T_low_* decrease, and then, *T_low_* gradually exceeds *T_up_*. Accordingly, *V_out_* starts to decrease and then increase again in an opposite direction. During the whole process, the output voltage is consistent with the temperature difference, the value of which basically stays above 0.5 V and reaches a maximum of 2 V.

**Figure 17:**
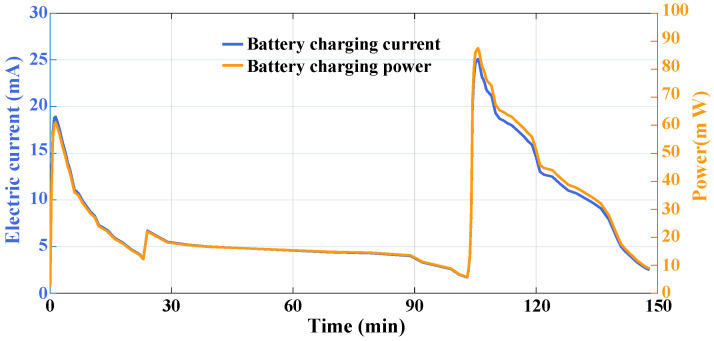
Energy recovery results.

The output voltage of the thermoelectric generator is connected to the power management unit of the multi-parameter monitoring system. By measuring the working current of the system when powered by the energy self-supply module, the output power can be calculated, as shown in Figure 17. The maximum energy harvest power occurs when the temperature difference between *T_low_* and *T_up_* reaches the maximum value. At this time, the maximum current and power are 25.1 mA and 87.5 mW, respectively, and the maximum power value is much higher than the power consumption value of the system. According to the measurement results in Figure 16, the total energy that can be recovered in one flight mission is 296.4 J, which can allow the system to operate for 11 h. Therefore, with the energy self-supply module, the multi-parameter monitoring system is capable of continuously working onboard without an external energy supply.

### 5.4. Composite Unmanned Aerial Vehicle Monitoring Verification

In this section, a composite unmanned aerial vehicle (UAV) wing is adopted to deploy the developed system, of which the multi-parameter monitoring and energy self-supply abilities are further verified.

#### 5.4.1. Experimental Setup

**Figure 18:**
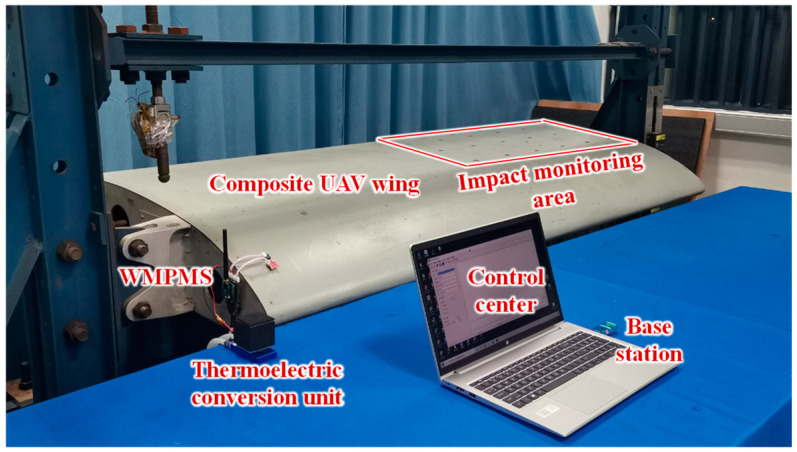
Multi-parameter monitoring of the composite UAV wing.

**Figure 19:**
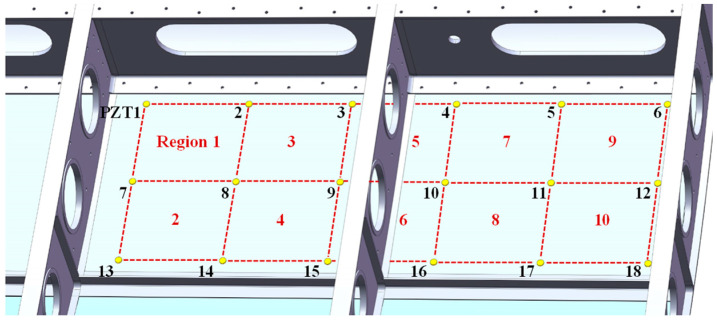
PZT placement on the impact monitoring regions.

The experimental setup of the multi-parameter monitoring of the composite UAV wing is shown in Figure 18. The multi-parameter monitoring system is fixed on the wing rib and used to monitor impact events and the environment of the wing structure. Figure 19 shows the PZT placement in the impact monitoring area, which contains 18 PZTs and forms 10 monitoring regions. The PZTs are attached to the inner surface of the wing, surrounded by complex structures like the wing rib, stiffener, and wing spar. The 10 regions are labeled from region 1 to region 10, and they are the same size, 170 mm × 150 mm. During the experiment, the system is woken up every 10 s for environment monitoring and is triggered to respond whenever an impact occurs. For environment monitoring, it should be noted that only temperature, humidity, and air pressure are monitored, and vibration is not considered in this experiment due to the limitations of the experimental setup.

In addition, in order to verify the energy self-supply ability, a TEC is also adopted to simulate temperature variation at the beginning of the experiment. By simulating the temperature variation during the take-off/landing stage of the aircraft, the temperature of the energy self-supply module is controlled to vary from 30 °C to −10 °C and then back to 30 °C again. During this process, part of the harvested thermal energy directly powers the multi-parameter monitoring system and the other part is stored in the rechargeable battery. After the energy self-supply module stops working, the rechargeable battery is used to continuously power the system.

#### 5.4.2. Experimental Verification Results

To verify the ability of impact monitoring, 20 impact events are conducted in every region. Based on the digital sequences obtained by the multi-parameter monitoring system, the *RWS* value of each PZT and then each region can be calculated. Therefore, the impact region can be identified. Table 4 gives out the impact monitoring results of every region compared with the FPGA-based digital impact monitoring system developed in the early stage. The impact monitoring accuracy of the composite structures has been found to increase from 86% to 96% [37].

In addition, thermocouples, hygrometers, and barometers are also adopted to verify the environment monitoring results of the multi-parameter monitoring system. Table 5 presents the typical comparison results of the temperature, humidity, and air pressure of the UAV wing. It can be seen from the table that there is good agreement between the monitoring results of the system and the adopted sensors.

However, the above verification work is carried out under ideal laboratory conditions. For example, for the verification of impact monitoring, the influence of external vibration sources (such as flight airflow, engines, etc.) on the impact signal is not considered. And the monitoring of service environment parameters is also different from the actual harsh environment of the aircraft. Therefore, this experiment only verifies the effectiveness of the proposed monitoring method and the developed monitoring system applied to the real aircraft skin structure. In order to enable the practical application of the aircraft skin, it is necessary to optimize the performance and stability of the monitoring system in the future, for example, by using high-performance devices to improve the sampling rate of the system, increasing the number of sensing channels, improving the anti-interference capability of the hardware, and accordingly optimizing the monitoring algorithm. The system needs to be functionally verified in more environments.

## 6. Conclusions

Aiming at ASS, this paper proposes an aircraft multi-parameter monitoring method, including a multi-parameter monitoring architecture and the corresponding all-digital multi-parameter monitoring method. Based on the proposed methods, a miniaturized and ultra-low-power WMPMS is developed, which is capable of monitoring the structure impact and service environment, including vibration, temperature, humidity, and air pressure. The size of the system is just 45 mm × 50 mm × 30 mm, and the power consumption is only 7.59 mW. In addition, a TEG-based energy self-supply module is also developed and is able to power the system, continuously working onboard for 11 h without an external energy supply. A series of verification experiments on a composite UAV wing were carried out to show the multi-parameter monitoring function of the system, as well as its power consumption and self-power supply ability. However, considering the practical aviation applications, there still exist the following issues that need to be further studied in the ongoing research.

(1) The monitoring system developed in this paper is only in the stage of completing the method and function verification. In the future, for more complex monitoring environments, whether the performance and reliability of the system can meet the monitoring requirements needs to be further tested, for example, by testing for a larger data processing capacity and higher data transmission speed.
(2) At present, the functional verification of monitoring systems is carried out in the ideal laboratory environment, without considering external interferences that may exist in the actual environment. In the future, the performance of the monitoring system should be evaluated and improved according to the actual application conditions. In terms of hardware, performance and stability should be considered, while in terms of software, operational efficiency and accuracy should be considered, as well as the iterative optimization of monitoring methods.
(3) As a structural health monitoring device with wireless communication capability, the developed WMPMS has scalability. An important research question that remains is how to organize multiple systems through network design to carry out multi-parameter data fusion monitoring and realize the large-scale, multi-point, and multi-directional monitoring of an aircraft.

## Figures and Tables

**Table 1:** *RWS* value of every PZT.

PZT Number	PZT 1	PZT 2	PZT 3	PZT 4	PZT 5	PZT 6	PZT 7	PZT 8	PZT 9
***RWS***	**2165**	**2394**	912	**2001**	**2801**	1003	528	603	342

**Table 2:** *RWS* value of every region.

Region Number	Region 1	Region 2	Region 3	Region 4
***RWS***	**9361**	7110	5933	4749

**Table 3:** Power consumption in different working states.

Working Mode	Voltage (V)	Current (mA)	Power Consumption (mW)	Duration (s) (Every 10 s)
Sleep	3.3	0.86	2.84	>9.5
Impact monitoring	3.3	1.65	5.45	<0.1
Vibration monitoring	3.3	1.16	3.83	10
Temperature and humidity monitoring	3.3	1.26	4.16	<0.1
Air pressure monitoring	3.3	1.21	3.99	<0.1
Wireless communication	3.3	28.06	92.6	<0.1

**Table 4:** Impact monitoring results on the composite UVA wing.

Impact Regions	Impact Times	Correct Location Times	Accuracy
1	20	20	100%
2	20	19	95%
3	20	20	100%
4	20	20	100%
5	20	19	95%
6	20	18	90%
7	20	18	90%
8	20	19	95%
9	20	20	100%
10	20	19	95%

**Table 5:** Digital sensor multi-parameter monitoring results.

Typical Time	Temperature Monitoring (°C)		Humidity Monitoring (%RH)		Air Pressure Monitoring (Pa)	
	System	Thermocouples	System	Hygrometers	System	Barometers
1	22.4	22.0	26.0	26.3	102,895	102,890
2	22.5	22.4	26.3	26.1	102,899	102,900
3	22.0	21.7	26.3	26.2	102,892	102,890
4	21.7	21.3	26.2	26.0	102,890	102,880
5	21.5	21.5	26.0	26.1	102,887	102,880
6	21.3	21.7	25.7	26.0	102,885	102,890
7	20.7	21.0	25.4	25.9	102,864	102,880
8	20.4	20.8	25.7	25.7	102,865	102,870
9	20.6	20.7	25.6	25.5	102,855	102,870
10	20.8	21.0	25.2	25.6	102,872	102,880

## Data Availability

The original contributions presented in this study are included in the article. Further inquiries can be directed to the corresponding authors.

## References

[1] McEvoy M.A., Correll N. (2015). Materials that couple sensing, actuation, computation, and communication. Science.

[2] Ren H.L., Yang X.D., Wang Z.H., Xu X.G., Wang R., Ge Q., Xiong Y. (2022). Smart structures with embedded flexible sensors fabricated by fused deposition modeling-based multimaterial 3D printing. Int. J. Smart Nano Mater..

[3] Wang Y., Hu S.G., Xiong T., Huang Y.A., Qiu L. (2022). Recent progress in aircraft smart skin for structural health monitoring. Struct. Health Monit..

[4] Dey S., Bhattacharyya R., Sarma S.E., Karmakar N.C. (2020). A novel “smart skin” sensor for chipless RFID-based structural health monitoring applications. IEEE Internet Things J..

[5] Yang Y., Chiesura G., Plovie B., Vervust T., Luyckx G., Degrieck J., Sekitani T., Vanfleteren J. (2018). Design and integration of flexible sensor matrix for in situ monitoring of polymer composites. ACS Sens..

[6] Suzuki Y., Suzuki T., Todoroki A., Mizutani T. (2014). Smart lightning protection skin for real-time load monitoring of composite aircraft structures under multiple impacts. Compos. Part A Appl. Sci. Manuf..

[7] Wang C., Dudley K., Szatkowski G. Open circuit resonant (SansEC) sensor for composite damage detection and diagnosis in aircraft lightning environments. Proceedings of the 4th AIAA Atmospheric and Space Environments Conference.

[8] Lin M., Chang F.K. (2002). The manufacture of composite structures with a built-in network of piezoceramics. Compos. Sci. Technol..

[9] Ren Y.Q., Zhang S.F., Yuan S.F., Qiu L. (2023). In-situ integration and performance verification of large-scale PZT network for composite aerospace structure. Smart Mater. Struct..

[10] Xiong W.N., Zhu C., Guo D.L., Hou C., Yang Z.X., Xu Z.Y., Qiu L., Yang H., Li K., Huang Y.A. (2021). Bio-inspired, intelligent flexible sensing skin for multifunctional flying perception. Nano Energy.

[11] Wang Y., Qiu L., Luo Y.J., Ding R. (2021). A stretchable and large-scale guided wave sensor network for aircraft smart skin of structural health monitoring. Struct. Health Monit..

[12] Kopsaftopoulos F., Nardari R., Li Y.H., Chang F.K. (2018). A stochastic global identification framework for aerospace structures operating under varying flight states. Mech. Syst. Signal Process..

[13] Sharp N., Kuntz A., Brubaker C., Amos S., Gao W., Gupta G., Mohite A., Farrar C., Mascareñas D. (2014). A bio-inspired asynchronous skin system for crack detection applications. Smart Mater. Struct..

[14] Zhao D., Liu T., Zhang M., Liang R., Wang B. (2012). Fabrication and characterization of aerosol-jet printed strain sensors for multifunctional composite structures. Smart Mater. Struct..

[15] Blumenthal T., Fratello V., Nino G., Ritala K. Conformal printing of sensors on 3D and flexible surfaces using aerosol jet deposition. Proceedings of the SPIE Smart Structures and Materials + Nondestructive Evaluation and Health Monitoring.

[16] Qiu L., Lin X.D., Yuan S.F., Shi W.L. (2020). A lightweight system with ultralow-power consumption for online continuous impact monitoring of aerospace vehicle structures. IEEE Trans. Ind. Electron..

[17] Krichen D., Abdallah W., Boudriga N. (2017). On the design of an embedded wireless sensor network for aircraft vibration monitoring using efficient game theoretic based MAC protocol. Ad Hoc Netw..

[18] Kim J.H., Park Y., Kim Y.Y., Shrestha P., Kim C.G. (2015). Aircraft health and usage monitoring system for in-flight strain measurement of a wing structure. Smart Mater. Struct..

[19] Kwon H., Park Y., Kim J.H., Kim C.G. (2019). Embedded fiber Bragg grating sensor–based wing load monitoring system for composite aircraft. Struct. Health Monit..

[20] Jiang X., Li Y.C., Wang J., Li J.C. (2014). Electromechanical modeling and experimental analysis of a compression-based piezoelectric vibration energy harvester. Int. J. Smart Nano Mater..

[21] Gao X.Z., Hou Z.X., Guo Z., Chen X.Q. (2015). Reviews of methods to extract and store energy for solar-powered aircraft. Renew. Sust. Energ. Rev..

[22] Junior O.H.A., Maran A.L.O., Henao N.C. (2018). A review of the development and applications of thermoelectric microgenerators for energy harvesting. Renew. Sust. Energ. Rev..

[23] Allmen L.V., Bailleul G., Becker T., Decotignie J.D., Kiziroglou M.E., Leroux C., Mitcheson P.D., Müller J., Piguet D., Toh T.T. (2017). Aircraft strain WSN powered by heat storage harvesting. IEEE Trans. Ind. Electron..

[24] Elefsiniotis A., Becker T., Schmid U. (2014). Thermoelectric energy harvesting using phase change materials (PCMs) in high temperature environments in aircraft. J. Electron. Mater..

[25] Samson D., Kluge M., Becker T., Schmid U. (2011). Wireless sensor node powered by aircraft specific thermoelectric energy harvesting. Sens. Actuators A Phys..

[26] Featherston C.A., Holford K.M., Waring G. (2009). Thermoelectric energy harvesting for wireless sensor systems in aircraft. Key Eng. Mater..

[27] Fu H.L., Khodaei Z.S., Aliabadi M.H.F. (2018). An event-triggered energy-efficient wireless structural health monitoring system for impact detection in composite airframes. IEEE Internet Things J..

[28] Abbas M., Shafiee M. (2018). Structural health monitoring (SHM) and determination of surface defects in large metallic structures using ultrasonic guided waves. Sensors.

[29] Capineri L., Bulletti A. (2021). Ultrasonic guided-waves sensors and integrated structural health monitoring systems for impact detection and localization: A review. Sensors.

[30] Muller A., Robertson-Welsh B., Gaydecki P., Gresil M., Soutis C. (2017). Structural health monitoring using lamb wave reflections and total focusing method for image reconstruction. Appl. Compos. Mater..

[31] Muttillo M., Stornelli V., Alaggio R., Paolucci R., Battista L.D., Rubeis T., Ferri G. (2020). Structural health monitoring: An IoT sensor system for structural damage indicator evaluation. Sensors.

[32] Aleman M.A., Gopalarathnam A., Granlund K. (2022). Novel Surface Flow-Reversal Sensor Applied to Detection of Airfoil Stall. J. Aircr..

[33] Nguyen V., Dugenske A. (2018). An I2C based architecture for monitoring legacy manufacturing equipment. Manuf. Lett..

[34] Yuan S.F., Liu P.P., Qiu L. (2012). A miniaturized composite impact monitor and its evaluation research. Sens. Actuators A Phys..

[35] Gao S., Dai X., Hang Y., Guo Y., Ji Q. (2018). Airborne wireless sensor networks for airplane monitoring system. Wirel. Commun. Mob. Comput..

[36] Nardo M.D., Yu H. (2021). Intelligent ventilation systems in mining engineering: Is ZigBee WSN technology the best choice?. Appl. Syst. Innov..

[37] Qiu L., Yuan S.F., Liu P.P., Qian W.F. (2013). Design of an All-Digital Impact Monitoring System for Large-Scale Composite Structures. IEEE Trans. Instrum. Meas..

